# Anti-neutrophil Cytoplasmic Antibody-Associated Glomerulonephritis Secondary to Hydralazine: A Case Report

**DOI:** 10.7759/cureus.32603

**Published:** 2022-12-16

**Authors:** Ahmed Abdullah, Muhammad Saad Anwar, Maria Ijaz, Muhammad Sikandar Karim, Raquel Rosen, Syed Rizwan A Bokhari

**Affiliations:** 1 Internal Medicine, California Institute of Behavioral Neurosciences & Psychology, Fairfield, USA; 2 Neurology, AINeuroCare, Dallas, USA; 3 Pediatrics, Al Aleem Medical College/Gulab Devi Chest Hospital, Lahore, PAK; 4 Internal Medicine, Allama Iqbal Medical College/ Jinnah Hospital Lahore, Lahore, PAK; 5 Research, California Institute of Behavioral Neurosciences & Psychology, Fairfield, USA; 6 Nephrology, Bassett Medical Center, Cooperstown, USA; 7 Nephrology, Tulane University School of Medicine, New Orleans, USA

**Keywords:** c anca, p-anca, antineutrophil cytoplasmic antibody (anca) associated vasculitis (aav), hydralazine-induced vasculitis, pauci-immune crescentic glomerulonephritis

## Abstract

Hydralazine is a vasodilator used in the treatment of resistant hypertension. It is a safe and widely used antihypertensive medicine. Its common adverse effects include headache, rebound tachycardia, fluid retention, and angina. It is a rare cause of anti-neutrophil cytoplasmic antibody (ANCA) associated vasculitis (AAV) with pulmonary and renal involvement. We report a case of a 74-year-old woman, with over eight years of use of hydralazine, who presented to the hospital with shortness of breath and cough. Blood work revealed deranged renal function with high creatinine levels. Serology workup was positive for anti-histone antibodies (AHA), anti-nuclear antibodies (ANA), myeloperoxidase (MPO) ANCA and proteinase-3 (PR-3) ANCA. Renal biopsy showed diffusely flattened tubular epithelium, focal micro vesicular degeneration, and focal loss of the brush border of the proximal tubular epithelium. Hydralazine was stopped and the patient was treated with corticosteroids, resulting in the resolution of her kidney injury.

## Introduction

Vasculitis is defined as inflammation of blood vessels. Anti-neutrophil cytoplasmic antibody (ANCA) production is a common risk factor for vasculitis. ANCA-associated vasculitis (AAV) is an autoimmune disease leading to systemic vascular inflammation with positive ANCA. Vascular inflammation can manifest systemically or can be limited to specific organs, e.g., kidneys or lungs [1]. AAV can cause a range of renal pathology including raised creatinine levels, proteinuria, hematuria, and glomerulonephritis. A kidney biopsy often reveals crescentic glomerulonephritis [2]. AAV can occur secondary to different medicines including propylthiouracil, thiamazole, sofosbuvir, minocycline, carbimazole, mirabegron, tumor necrosis factor ɑ inhibitors, and hydralazine. Hydralazine is used for the management of refractory hypertension and hypertension in pregnancy and heart failure [3]. Hydralazine is also associated with drug-induced lupus (DIL). DIL does not commonly involve the kidneys, but when present, it can have a similar presentation to AAV. However, ANCA levels in DIL remain normal. Therefore, it is important to undergo ANCA evaluation for accurate diagnosis. Elevated levels of ANCAs are diagnostic of AAV [4]. Hydralazine-associated vasculitis is well described in the literature but remains a rare occurrence. Here, we present a rare case of Hydralazine-Induced Glomerulonephritis (HIG) with positive serologies for both AAV and DIL.

## Case presentation

A 74-year-old female patient with a history of hypertension, chronic obstructive pulmonary disease, asthma, coronary artery disease, transient ischemic attack, stage III chronic kidney disease, and multiple sclerosis presented to the hospital with a cough and shortness of breath for three days. Cough and shortness of breath had a gradual onset and worsened with time. The cough was constant, dry, and linked with wheezing episodes, although it was not accompanied by nausea or vomiting.

A review of systems was significant for generalized weakness and malaise. She denied any history of black or tarry stool, hemoptysis, or hematemesis. The family history was unremarkable. The patient denied using alcohol or illegal drugs but had a 25-pack-year smoking history. Her home medications included amlodipine, aspirin, bisacodyl, calcium carbonate, carvedilol, clonidine, clopidogrel, docusate sodium, famotidine, gabapentin, guaifenesin, isosorbide mononitrate, latanoprost, loratadine and hydralazine.

Other than tachycardia (pulse 105/min) patient's vitals were unremarkable. A comprehensive physical examination revealed bilateral lower extremity pitting edema. The biochemical investigation showed anemia with a hemoglobin (Hb) 5.9 mg/dL with mean corpuscular volume (MCV) of 78 fL with normal white blood cells and platelet count. Subsequent screening esophagogastroduodenoscopy and colonoscopy were unremarkable. D-dimer was 3400 mg/L and brain natriuretic peptide (BNP) was 642 pg/mL. Renal function tests showed increased creatine levels, up to 2.6 from her baseline of 1.2-1.5 since 2009. The urine dipstick was positive for blood and protein. Urinalysis showed 25-30 RBCs (> 50% dysmorphic) without casts. Urine microscopy was significant for 25-30 RBCs with more than 50% dysmorphic RBCs. 24-hour urine protein was 4.2 g with total serum proteins at 6.6 mg/dL and serum albumin at 3.5 mg/dL. Chest x-ray was concerning for left-sided basal opacity. Non-contrast computed tomography (CT) scan of the chest showed a left basal consolidation and multifocal patchy and nodular opacities. Ventilation-perfusion scan demonstrated a low probability of pulmonary embolism. She also tested positive for influenza A. The patient was admitted for pneumonia and influenza and broad-spectrum antibiotics including meropenem, vancomycin, and Tamiflu were initiated along with a transfusion of three units of packed RBCs.

**Figure 1:**
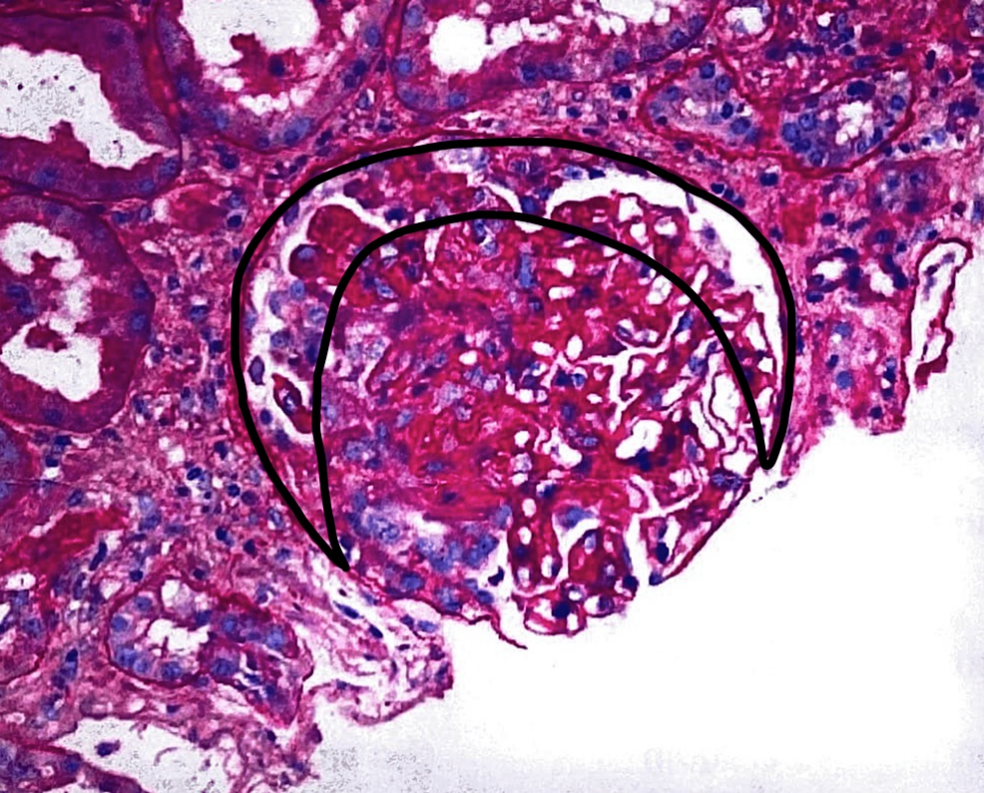
Necrotizing and crescentic glomerulonephritis Original biopsy figure.

**Figure 2:**
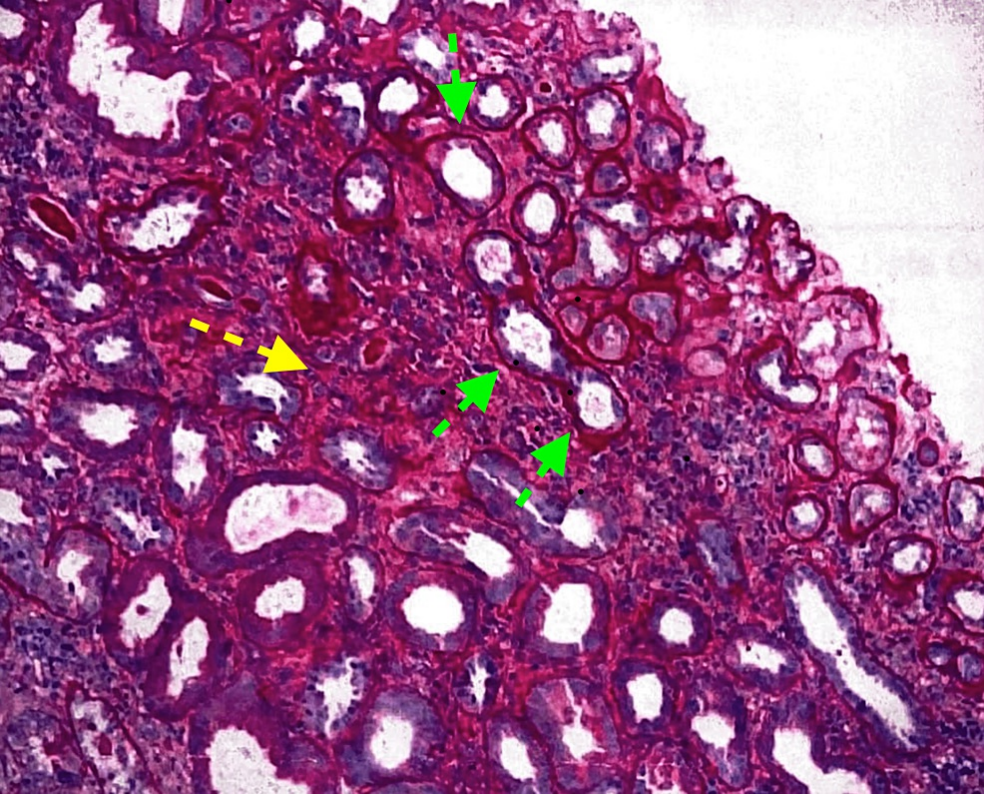
Tubular atrophy and interstitial fibrosis Original biopsy figure. Yellow arrow → interstitial fibrosis, green arrow → tubular atrophy

Subsequent renal ultrasound showed a normal bilateral kidney structure with no mass, hydronephrosis, calculus, or perinephric collection. A nephritic syndrome workup was done which showed C-reactive protein (CRP) 3.6 mg/dL, erythrocyte sedimentation rate (ESR) 54 mm/hr, normal complement 3 (C3) and low complement 4 (C4) levels. Hepatitis B surface antigen (HbsAg), hepatitis C antibody and human immunodeficiency virus (HIV) serologies, rheumatoid factor, and serum protein electrophoresis for monoclonal light chains were negative. Serology was positive for the anti-nuclear antibody (ANA), anti-histone antibody (AHA), proteinase-3 (PR-3), and myeloperoxidase (MPO) ANCA. Hydralazine was stopped due to suspicion of glomerulonephritis due to hydralazine and a kidney biopsy was performed. Microscopic examination of slides showed diffusely flattened tubular epithelium, focal microvesicular degeneration, and focal loss of the brush border of the proximal tubular epithelium. A few tubules contained granular casts, periodic-acid-Schiff (PAS) staining-positive hyaline casts, and RBC casts in various stages of degeneration. The interstitium had mild interstitial inflammation in areas of atrophy. Diagnosis of necrotizing crescentic glomerulonephritis (Figure 1) with tubular atrophy and interstitial fibrosis (Figure 2) was made based on clinical and pathological findings.

The patient was started on intravenous solumedrol which resulted in rapid improvement of edema and normalization of creatinine level over one week. Rituximab was offered as a first-line treatment due to a lower risk of adverse effects as compared to cyclophosphamide and steroid but was refused by the patient. She was discharged from the hospital on 60 mg of oral prednisone for two weeks.

## Discussion

Since 1951, hydralazine has been used as a treatment option for refractory hypertension. It is a common cause of DIL, glomerulonephritis, and AAV [2]. Other drug-induced causes of AAV include montelukast, minocycline, rituximab, tumor necrosis factor-ɑ inhibitors (etanercept, infliximab, adalimumab, golimumab), and anti-thyroid drugs (methimazole, propylthiouracil, carbimazole, and benzyl thiouracil). Risk factors for drug-induced renal injury include old age (>60 years), female gender, diabetes, heart failure, and pre-existing renal insufficiency [2,5].

AAV is a rare form of vasculitis with 10-20 cases per million reported annually worldwide. AAV commonly affects the pulmonary and renal vessels and is associated with antibodies against MPO and PR-3, which are differentiated by diffuse or perinuclear cytoplasmic immunofluorescence staining patterns, respectively [6]. Pulmonary involvement is most associated with mortality [7]. AAV secondary to hydralazine is directly dependent on the duration and dose of hydralazine use. The use of hydralazine for more than three years can have an annual incidence of up to 5.4% and 10.4% in patients taking 100mg/day and 200mg/day, respectively [8].

Clinically, drug-induced AAV presents in a similar fashion to idiopathic AAV, affecting the skin, kidneys, and lungs. Similarly, lupus nephritis and hydralazine-induced vasculitis can have an overlapping presentation of elevated serum creatinine and increased anti-double-stranded DNA antibody titers and low complement levels. In such cases, a biopsy typically confirms the diagnosis by distinguishing between the immunofluorescence patterns of lupus nephritis and hydralazine-induced vasculitis [4,6]. Commonly, patients using hydralazine can have symptoms of glomerulonephritis as well as positive ANA and AHA [9]. Even in the absence of any symptoms of systemic lupus erythematosus, ANA and AHA are positive in 5%-8% of patients using hydralazine. Therefore, in the absence of other symptoms, positive AHA is not diagnostic of DIL [10,11]. Patients with HIG have severe progression compared to those with DIL because of vasculitis of vessels of kidneys, and therefore require aggressive immunosuppressant treatment [5].

It is still under debate as to how hydralazine triggers an immune response. The most widely accepted theory is that hydralazine builds up in neutrophil cytoplasmic granules and then binds to MPO, causing the release of cytotoxic chemicals and cell death. Antigen-presenting cells are eventually exposed to usually hidden antigens as a result, and ANCA and ANA are then produced. Ultrasound-guided percutaneous renal biopsy, which has been shown to have a low risk of complications such as hemorrhage, should be used to aid in the diagnosis and treatment of AAV [7]. Pauci-immune glomerulonephritis patients frequently have quickly progressing glomerulonephritis with brown urine suggestive of glomerular hematuria. Proteinuria and renal insufficiency are additional findings. Systemic vasculitis syndrome's extra-renal presentation often includes fever, malaise, arthralgia, myalgia, and weight loss [12].

Here, we present a case of HIG in an elderly female patient with acute kidney injury on chronic kidney disease with long-term hydralazine use. The rapid onset of acute glomerulonephritis, positive ANCA antibodies, and resolution of creatinine levels to baseline after discontinuation of hydralazine and characteristic features on kidney biopsy confirmed the diagnosis of glomerulonephritis due to hydralazine. Other possible diagnoses included lupus nephritis and anti-glomerular basement disease which were excluded by clinical evaluation. Although multi-organ involvement including kidney dysfunction is common in AAV, isolated involvement of kidneys without symptoms is a rare occurrence. In such cases, renal histopathology demonstrates diffuse sclerosing glomerulonephritis with fibrous crescents [13].

Management of HIG depends upon the patient's risk factors. Immediate discontinuation of hydralazine is the primary step, which usually leads to the resolution of symptoms in mild cases. Severe presentations with kidney or lung involvement usually require the use of immunosuppressive therapies including steroids, cyclophosphamide, or rituximab [6,14,15]. The ANCA usually remains positive even after treatment with immunosuppressants despite the resolution of symptoms. Hemodialysis may be necessary acutely for instances of pulmonary edema, hyperkalemia, or other acute indications. There are reports of patients developing end-stage kidney disease requiring maintenance hemodialysis. In reported cases, renal outcomes for this disease have been variable, with one case study claiming full recovery in all patients and another reporting that one-third to half of the patients still needed long-term hemodialysis following immunosuppressive treatment [5,8,9].

## Conclusions

Hydralazine is a common cause of AAV and DIL. In addition to positive serologies for ANA and AHA, MPO and PR-3 ANCA are also present in drug-induced vasculitis. Although hydralazine-induced vasculitis commonly involves the pulmonary and renal systems, isolated renal involvement can also occur. Discontinuation of hydralazine and the use of immune modulators is the mainstay treatment.
